# Wearable Sensors Reveal Head–Sternum Dissociation as a Latent Deficit in Active Aging

**DOI:** 10.3390/s26072125

**Published:** 2026-03-29

**Authors:** András Salamon, Gabriella Császár

**Affiliations:** 1 Doctoral School of Health Sciences, Faculty of Health Sciences, University of Pécs, 7622 Pécs, Hungary; 2 Department of Physiotherapy, Institute of Physiotherapy and Sport Science, Faculty of Health Sciences, University of Pécs, 8900 Zalaegerszeg, Hungary

**Keywords:** postural control, head–sternum dissociation, wearable sensors, IMU, aging, neuro-mechanical phenotypes, dance, early detection

## Abstract

**Highlights:**

**What are the main findings?**

- Early Detection of Latent Deficits: The novel inertial measurement unit (IMU)-based Head–Sternum Dissociation Index (HSDI) detects subclinical postural decline before standard functional mobility tests (e.g., Timed Up and Go) show impairment.
- Identification of a 55-Year Breakpoint: Kinematic analysis reveals a critical statistical threshold at age 55, marking the generalized collapse of compensatory sensorimotor mechanisms.

**What are the implications of the main findings?**

- Discovery of Sex-Specific Aging Profiles: Postural control degradation is sex-dependent; males adopt a rigid “binary” stiffening strategy, whereas females rely on a wider physiological buffer before shifting into chaotic instability.
- Neuroprotective Impact of Rhythm: Middle-aged adults with recreational rhythmic training (dance) successfully neutralize age-related kinematic decay, maintaining youthful head–trunk coordination.

**Abstract:**

Background: Traditional functional mobility assessments often fail to detect subclinical postural decline in active aging populations. This study introduces the Head–Sternum Dissociation Index as a novel digital biomarker to identify latent sensorimotor deficits before macroscopic balance failure occurs. Methods: Ninety-four participants (Young, Middle-Aged Civil, Middle-Aged Dancers, and Older Adults) performed instrumented limits of stability tasks, specifically functional and lateral reach tests, utilizing a three-sensor inertial measurement unit configuration. Postural strategies were quantified via the Head–Sternum Dissociation Index and the peak ratio of corrective micro-movements, validating the sensor output against a gold-standard force platform. Results: A significant kinematic breakpoint in postural control was identified at age 55 (*p* < 0.001). However, Middle-Aged Civilians exhibited early kinematic divergence despite maintaining normal Timed Up and Go test performance. Receiver operating characteristic analysis revealed distinct, sex-specific physiological limits: aging males predominantly adopted a rigid “Stiffness” strategy (peak ratio ≤ 1.15, head–sternum dissociation threshold > 0.63°), while females utilized a broader, more permissive “Continuous” strategy (head–sternum dissociation threshold > 0.31°). Notably, recreational rhythmic training (dance) completely neutralized this age-related decay, with middle-aged dancers maintaining highly efficient, youthful stabilization profiles (Cohen’s d = 2.20). Conclusions: The Head–Sternum Dissociation Index, combined with relative corrective frequency, successfully phenotypes early sensorimotor erosion. These findings advocate for the integration of sex-specific kinematic screening into primary care, allowing clinicians to prescribe targeted interventions well before clinical fall risk manifests.

## 1. Introduction

### 1.1. The Paradox of Functional Aging

Maintaining functional mobility is widely recognized as a cornerstone of healthy and successful aging. However, a significant clinical paradox exists: many older individuals who perform within normal limits on conventional mobility assessments—such as the Timed Up and Go (TUG) [1,2]—already exhibit significant underlying neural and biomechanical decline. While the TUG remains a reliable and valid tool for clinical screening across the lifespan, its ability to predict future falls in community-dwelling older adults has been questioned due to a potential ceiling effect in relatively active populations [3].

This phenomenon can be described as a “functional mask,” where the central nervous system successfully employs compensatory mechanisms to maintain gross motor performance, thereby obscuring the progressive erosion of postural control. Consequently, relying solely on time-based or categorical clinical outcomes may lead to a delayed diagnosis of fall risk. To bridge this gap, there is a critical need to move beyond simple timing towards instrumented assessments (instrumented Timed Up and Go [iTUG]) that can quantify the quality of movement and detect the early, subclinical stages of sensorimotor impairment [4,5]. The integration of wearable inertial measurement units (IMUs) offers a promising solution, allowing for the real-time monitoring of postural strategies and the identification of latent deficits that remain invisible to the naked eye or traditional stop-watch-based metrics.

### 1.2. The Biomechanics of Head Stability

The stabilization of the head in space is one of the most critical tasks of the human postural control system, as it provides a steady reference frame for the integration of visual and vestibular inputs [6]. Maintaining an upright and stable head position is not merely a local neck-control task but a global challenge that requires the precise coordination of multiple body segments to preserve equilibrium during both static and dynamic activities [7].

As individuals age, the precision and reliability of these sensory systems gradually decline. To compensate for reduced proprioceptive acuity and vestibular feedback, the central nervous system often shifts towards a “stiffening” strategy. This involves an increased co-contraction of the neck and trunk musculature, effectively “locking” the head to the torso. While this strategy may provide a temporary sense of stability and reduce the relative motion between segments, it significantly limits the degrees of freedom available for adaptive postural responses. Previous research has demonstrated that older adults exhibit significantly altered coordination strategies compared to younger individuals, particularly when visual input is compromised, leading to greater head instability during locomotor tasks [8]. Understanding these kinematic shifts is vital for identifying the transition from flexible, proactive control to the rigid, reactive strategies that characterize an increased risk of falls.

### 1.3. Wearable Technology and Kinematic Biomarkers

The emergence of wearable IMUs has revolutionized the field of motion analysis by providing high-resolution kinematic data outside of traditional, expensive laboratory settings. Unlike gold-standard optical motion capture systems, which are constrained by fixed camera volumes and complex calibration procedures, wearable sensors allow for the objective quantification of fall risk in ecologically valid, everyday environments [9]. This technological shift is particularly relevant for geriatric assessments, where the ability to monitor subtle changes in gait and postural sway over time—rather than relying on a single clinical snapshot—can lead to more timely and personalized interventions.

Kinematic biomarkers derived from these sensors offer a deeper insight into the quality of motor control than traditional outcome measures. While a stopwatch-based test might confirm that an individual can still complete a task, IMU-based metrics can reveal the underlying “how”—detecting increased accelerations, terminal sway, or altered segment coordination that often precede functional failure and actual falls [10]. Despite these advantages, the clinical adoption of wearable technology requires robust validation against established standards for testing postural function [11]. Furthermore, the practical use of such technology, including simpler tools like inclinometers, has shown promise in real-time balance control monitoring, provided they are validated against professional biomechanical metrics [12]. Recent advancements in joint kinematics estimation using magnetic and inertial sensing modules further emphasize the need for precise algorithmic approaches to ensure reliability in diverse clinical populations [13].

### 1.4. The Current Study: The Head–Sternum Dissociation Index Approach

While traditional fall detection systems and basic kinematic metrics (such as isolated angular displacement) have been extensively reviewed for their utility in older adults [14], they primarily focus on identifying macroscopic loss of balance or post-fall events. However, recent literature emphasizes the critical need for ‘digital biomarkers’ capable of detecting subclinical, latent postural deficits before a catastrophic balance failure occurs. Conventional metrics often fail to capture the qualitative shift from flexible, multi-segmental coordination to rigid, co-contractive buffering strategies. Therefore, the introduction of the Head–Sternum Dissociation Index (HSDI) is not merely exploratory, but a clear biomechanical imperative.

The primary objective of this study is to introduce and validate the HSDI as a sensitive kinematic biomarker for early postural decline. While the importance of multi-segmental coordination and the role of head stabilization have been conceptually established [6,7], the specific dissociation between the head and the upper torso remains an underexplored area in the context of subclinical aging. By focusing on the spatial relationship between these segments, we aim to bridge the gap between laboratory-grade static postural assessments [12] and portable, real-world kinematic monitoring [13].

Building upon the necessity for objective metrics that transcend simple timing-based tests, we hypothesize that:

1. The IMU-derived kinematic metrics will demonstrate high concurrent validity when compared against gold-standard force platform metrics, providing a reliable proxy for center-of-pressure (COP) stability.
2. A statistical breakpoint in postural control can be identified around the age of 55, marking a significant transition in the age-related decline of head–sternum coordination.
3. Recreational rhythmic training (dance) serves as a powerful protective factor, allowing middle-aged and older individuals to maintain youthful head-stabilization strategies that are distinct from those of their age-matched peers.

By mapping these findings onto a Kinematic Stability Matrix, this research seeks to provide a clinical framework for identifying “at-risk” individuals decades before overt mobility limitations manifest.

## 2. Materials and Methods

### 2.1. Participants

A total of 94 participants were recruited for this cross-sectional study. Initial recruitment targeted three standard cohorts based on chronological age (Young: 18–25; Middle: 26–64; Elderly: >65). However, preliminary data analysis revealed a significant non-linear deterioration in kinematic stability starting at age 55. Consequently, a post-hoc re-stratification was performed to accurately capture this latent deficit. The final analysis groups were defined as:

- Young Adults (N = 30): Healthy university students (Age: 18–25 years).
- Middle-Aged Civil (N = 20): Adults with a sedentary or recreationally active lifestyle (Age: 26–54 years). This group (referred to as ‘Control’ in figures) consisted of individuals leading an active lifestyle but having no prior experience in rhythmic movements or formal dance training.
- Middle-Aged Dancers (N = 10): Experienced amateur folk and salsa dancers (Age: 26–54 years), serving as an active recreational control group. This cohort was specifically age-matched to the Middle-Aged Civil group to isolate the effects of sensorimotor training from chronological aging, acting as a “neuro-biomechanical gold standard” to establish the baseline for optimal multi-segmental stabilization.
- Older Adults (N = 34): Community-dwelling adults above the identified kinematic threshold (Age: ≥55 years).

#### 2.1.1. Inclusion and Exclusion Criteria

To ensure that the observed differences were due to aging and not pathology, strict exclusion criteria were applied. Participants were excluded if they had: (1) any history of neurological or vestibular disorders (e.g., stroke, Parkinson’s disease, vertigo); (2) orthopedic surgery or lower limb injury within the last 12 months; (3) significant musculoskeletal pain (VAS > 3/10) affecting gait; or (4) inability to walk 10 m independently without assistive devices.

#### 2.1.2. Ethics Statement

The study was conducted in accordance with the Declaration of Helsinki. The protocol was approved by the Institutional Review Board of ETT TUKEB (Approval No.: BM/7855-3/2025). All participants provided written informed consent prior to data collection.

### 2.2. Instrumentation and Sensor Setup

**Figure 1:**
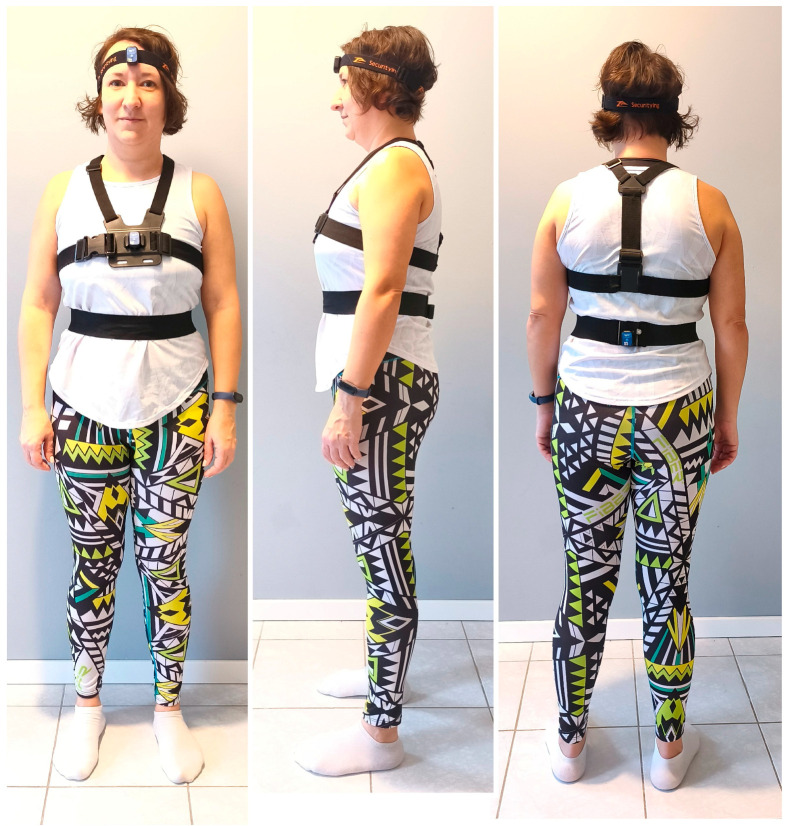
Sensor placement on Head, Sternum, and Lumbar spine (Written informed consent was obtained from the participant for the publication of this image).

To ensure high-fidelity kinematic data collection, we utilized a multi-sensor configuration:

- IMUs: Three wireless high-precision sensors (WitMotion WT9011DCL, WitMotion Shenzhen Co., Ltd., Shenzhen, China) were employed. Each unit contains a 9-axis motion tracking module (3-axis gyroscope, 3-axis accelerometer, 3-axis magnetometer) with an internal Kalman filter to output stable Euler angles at a sampling rate of 100 Hz via BLE 5.0, providing sufficient temporal resolution to capture high-frequency postural micro-adjustments and ‘stiffening’ behaviors.
- Sensor Placement: Sensors were secured using elastic Velcro straps (Nantong Junmao E-Commerce Co., Ltd., Nantong, China) to minimize soft-tissue artifacts at three anatomical landmarks (Figure 1):
- Reference System: A Zebris FDM-S force distribution platform (Zebris Medical GmbH, Isny im Allgäu, Germany) was used to record CoP displacement and force distribution parameters, serving as the “gold standard” for validation. The platform performed sampling at 100 Hz. The platform consists of 2560 capacitive sensors measuring vertical ground reaction forces.
- Following the placement of the IMU sensors, participants’ body height and the precise vertical distance of each sensor from the ground were measured using a Mileseey M120-B (Mileseey, City of Industry, CA, USA) high-precision laser distance meter. These measurements ensured that the Functional Reach Test (FRT) and Lateral Reach Test (LRT) values could be normalized to each participant’s height, allowing for an anatomically unbiased comparison of reach performance across the cohorts.

### 2.3. Measurement Protocol

The study followed a standardized concurrent validity protocol. Participants performed the tests barefoot to eliminate shoe-related instability. Data collection followed a standardized custom protocol (internal designation: Mésure) designed to synchronize the IMU kinematics with the force platform data.

#### 2.3.1. Dynamic Zero-Position Calibration

Before each trial, participants stood motionless in a neutral, relaxed posture on the Zebris platform for 3–5 s. The orientation data averaged during this window served as the baseline (t_0) for the IMUs, correcting for individual spinal curvatures (e.g., thoracic kyphosis) and ensuring that subsequent angular data represented relative excursion, not absolute anatomy.

#### 2.3.2. Experimental Protocol

The measurement protocol consisted of three consecutive instrumented stability tasks performed on the Zebris platform. Each task was recorded for a duration of 10 s (resulting in a total analysis window of 30 s per participant) to capture the dynamic stability limits:

1. Instrumented Functional Reach Test (iFRT): Maximal forward reach maintained for 10 s.
2. Instrumented Lateral Reach Test (iLRT—Right): Maximal reach to the right side (10 s).
3. Instrumented Lateral Reach Test (iLRT—Left): Maximal reach to the left side (10 s).

Data from the three intervals were aggregated to calculate the total frequency of kinematic micro-adjustments (peaks). The sensors were secured using adjustable elastic straps, which provided consistent fixation throughout the entire protocol, effectively eliminating motion artifacts or sensor migration.

In addition, a standard TUG test was performed separately to assess functional mobility.

### 2.4. Data Processing

Raw sensor data processing, including signal fusion and kinematic feature extraction, was performed using MATLAB version R2023b (MathWorks Inc., Natick, MA, USA), while subsequent statistical analyses were executed via custom Python scripts. The WitMotion sensors employ an internal dynamic Kalman filter algorithm to fuse accelerometer and gyroscope data, minimizing drift and providing stable real-time Euler angle outputs (Roll, Pitch, Yaw). The manufacturer specifies a dynamic angle accuracy of 0.1° (X and Y axes). The kinematic signals were synchronized with the Zebris force platform data to ensure temporal alignment.

#### 2.4.1. Two-Stage Kinematic Filtering and Hysteresis Thresholding

**Figure 2:**
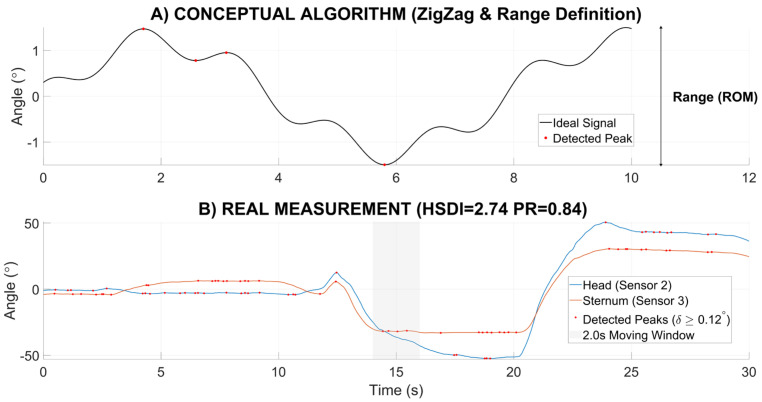
Kinematic signal processing and extraction of the HSDI. (**A**) Conceptual algorithm: Schematic representation of the two-stage discrete trend reversal (ZigZag) peak detection method. The algorithm identifies local extrema in the angular displacement waveform, representing deliberate postural micro-corrections in the ML plane. A minimum peak prominence threshold (δ = 0.12°) is applied to explicitly filter out high-frequency instrumental noise and physiological tremor. (**B**) Real measurement: Representative Kalman-filtered angular displacement data obtained from the wearable IMU sensors during an instrumented stability task. The moving window approach (w = 2.0 s) captures the spatial envelope (angular range) of the head and sternum. This spatial constraint is used to compute the continuous HSDI, providing a mathematically robust metric for inter-segmental rigidification without the integration drift associated with linear velocity calculations.

To quantify the dynamic corrective attempts of the postural system and to ensure maximum methodological rigor, we replaced standard frequency filtering with a discrete trend reversal algorithm (commonly known as the ZigZag method), implemented as a local extremum detection in MATLAB (Figure 2A). This formed the first stage of a two-stage filtering process designed to differentiate deliberate, neurally driven cervical micro-corrections from inherent physiological and instrumental noise.

In the first stage (spatial amplitude filtering), a corrective movement (Peak) in the medio-lateral (ML) plane was registered only if the angular displacement exceeded a specific noise-rejection threshold (δ), acting as a minimum peak prominence:

|x_current_ − x_last_peak_| ≥ δ

Based on empirical calibration—derived from static resting noise measurements of the IMU sensors—we set δ = 0.12°. This threshold effectively filters out physiological tremor and high-frequency sensor noise while preserving the macroscopic corrective adjustments characteristic of functional cervical motor control during functional stability tasks.

Second, a functional coupling threshold was established using the Peak Ratio (PR). Theoretically, a PR of 1.0 represents perfect kinematic coupling between the head and the trunk (a 1:1 correction ratio). However, based on standard biomechanical reliability metrics, the natural intra-subject Coefficient of Variation (CoV) and Minimal Detectable Change (MDC) in quiet standing typically approach 10–15% due to physiological artifacts such as respiratory mechanics, cardiovascular micro-vibrations, and anatomical slack [15,16,17]. Furthermore, from the perspective of dynamical systems theory, rigid inter-segmental coupling in human motor control operates within a flexible margin rather than maintaining absolute mathematical synchrony [18]. Therefore, a ±15% margin of error (0.85 ≤ PR ≤ 1.15) was established a priori to define the ‘Coupled/Stiffness’ phenotype. This ensures that only PR values exceeding 1.15—where the frequency of cephalic micro-corrections significantly surpasses the baseline physiological noise—are classified as functionally independent neural strategies (i.e., Dissociation or Active Stabilization). This theoretical threshold was further validated empirically by our dataset: a percentile analysis revealed a natural statistical breakpoint, as the 25th percentile of the PR in the healthy, actively stabilizing young cohort was >1.20, effectively separating the ‘Active’ phenotype from the age-related ‘Stiff’ phenotype. The validity and reliability of utilizing estimated angular information from inertial sensors for evaluating trunk kinematics have been strongly supported by recent literature [19].

#### 2.4.2. Windowed HSDI

To account for the continuous nature of balance, the HSDI was calculated using a moving window approach (Window Size w = 2.0 s, Figure 2B), rather than a single static snapshot. For each window, the range of motion (ROM) of the sternum was subtracted from the head:

HSDI_win_ = mean(Range_Head_) − mean(Range_Sternum_)

Interpretation

- HSDI ≈ 0: Indicates a “Block Strategy” (head and trunk move as a rigid unit), typical of the “Stiffness” phenotype found in high-risk males.
- HSDI > 0 (Positive): Indicates “Instability” (head moves significantly more than the trunk), typical of the “Dissociation Strategy” found in aging females.
- HSDI < 0 (Negative): Indicates “Active Stabilization” (head remains stable despite trunk movement), typical of the “Statue” or “Flow” strategies observed in dancers.

A key methodological feature is that the HSDI relies on unweighted orientation data, ensuring the index remains independent of individual anthropometric variations (e.g., neck length). Angular range was deliberately selected over velocity or acceleration metrics. Biomechanically, age-related rigidification manifests as a spatial constraint. Technically, while IMU-derived linear velocity and raw acceleration are highly susceptible to integration drift and movement-induced artifacts during dynamic reach tasks, the Kalman-filtered angular displacement provides the most robust and reliable metric for assessing these spatial boundaries. This approach allows for high-fidelity diagnostic output using only a three-sensor configuration, avoiding hardware-intensive calibration.

### 2.5. Statistical Analysis

An a priori power analysis was conducted using G*Power (version 3.1.9.6) to determine the minimum required sample size for the primary validation of the IMU system. For a bivariate correlation model, assuming a strong effect size (r = 0.50) with an alpha of 0.05 and a power of 0.80, a minimum of N = 21 participants was required. Our final recruited sample of N = 94 significantly exceeds this baseline. This deliberate oversampling was essential to ensure that after stratifying the data into sex-specific and age-related cohorts, the resulting subgroups remained sufficiently powered for meaningful comparative analysis. The group sizes ranged from a specialized cohort of *n* = 10 (recreational dancers) to a robust cohort of *n* = 34 (older adults), ensuring that even our most sensitive clinical comparisons were based on stable data distributions.

Raw sensor data preprocessing, including Kalman-filtering, signal smoothing, and the extraction of kinematic metrics (HSDI and PR), were performed using MATLAB version R2023b (MathWorks Inc., Natick, MA, USA). MATLAB was also utilized to conduct the iterative rolling-age-window breakpoint analysis and the Receiver Operating Characteristic (ROC) curve analysis to determine optimal kinematic thresholds.

Subsequent inferential and descriptive statistical analyses—including the assessment of data normality (Shapiro–Wilk test), group comparisons via one-way analysis of variance (ANOVA) with Tukey’s HSD post-hoc tests, and bivariate correlations—were carried out using Python version 3.9 (Python Software Foundation, Wilmington, DE, USA) with the pandas and SciPy libraries.

The age-group boundaries, specifically the 55-year cutoff for the older cohort, were not chosen arbitrarily but were determined through an objective, data-driven approach. An iterative rolling-age-window analysis (breakpoint analysis) identified age 55 as the statistical point where kinematic stability in the HSDI begins to deviate significantly from the middle-aged baseline. To ensure the robustness of this threshold, a sensitivity analysis was performed excluding the dancer cohort, and the 55-year breakpoint remained consistently significant (*p* < 0.001). By utilizing this physiologically-justified boundary, the study more accurately captures the transition from middle-adulthood to subclinical postural decline.

To evaluate differences between the re-stratified age cohorts (Young, Middle-Aged, Older Adults), a one-way ANOVA was conducted. Where significant main effects were found, post-hoc pairwise comparisons were performed using Tukey’s HSD test to identify specific group differences. The relationship between the IMU-derived HSDI metric and the gold-standard Zebris force platform parameters (Center of Pressure excursions) was assessed using Pearson’s correlation coefficient (r).

The critical age threshold (“breakpoint”) for HSDI deterioration was determined through an iterative analysis of variance across rolling age windows. Finally, Receiver Operating Characteristic (ROC) curve analysis was utilized to define normative reference values for identifying latent sensorimotor decline (Figure S3).

Due to significant kinematic differences between sexes, distinct cut-offs were established by maximizing the Youden Index (J = Sensitivity + Specificity − 1) to discriminate between the healthy control group (Young and Middle-Aged) and the high-risk Older Adult cohort:

- For Males: The optimal instability threshold was established at HSDI > 0.63° (Area Under the Curve (AUC) = 0.70 **, Youden’s J = 0.30).
- For Females: A lower, more sensitive threshold was identified at HSDI > 0.31° (AUC = 0.83, Youden’s J = 0.65 **), reflecting their different biomechanical baseline.

## 3. Results

### 3.1. System Reliability and Kinematic Baseline (Nested Pilot Study)

To evaluate the test–retest reliability and measurement error of the HSDI and PR metrics independently from the primary cohort, a nested pilot study was conducted with 10 healthy, asymptomatic young adult females (mean age: 20.9 ± 0.5 years). Thirty-second continuous standing trials across two sessions were analyzed to establish a robust kinematic baseline. A paired t-test revealed no significant systematic bias between sessions for either the postural stability volume (HSDI; *p* = 0.392) or the compensatory strategy ratio (PR; *p* = 0.209), confirming the absence of learning or fatigue effects.

The PR metric demonstrated excellent reliability (ICC = 0.814) over the entire measurement window. To determine the absolute measurement error, Standard Error of Measurement (SEM) and Bland–Altman Limits of Agreement (LoA) were calculated. Following a standard Interquartile Range (IQR) filtering to remove severe sensor artifacts (*n* = 1 excluded), the device exhibited high precision: for HSDI, the SEM was 1.16° (LoA: [−3.11° to 3.29°]), while for PR, the SEM was 0.11 (LoA: [−0.25 to 0.35]). These robust reliability statistics confirm that the system is highly consistent in tracking human postural control and suitable for the detailed sub-phase kinematic analysis executed in the primary cohort.

### 3.2. Participant Characteristics

**Figure 3:**
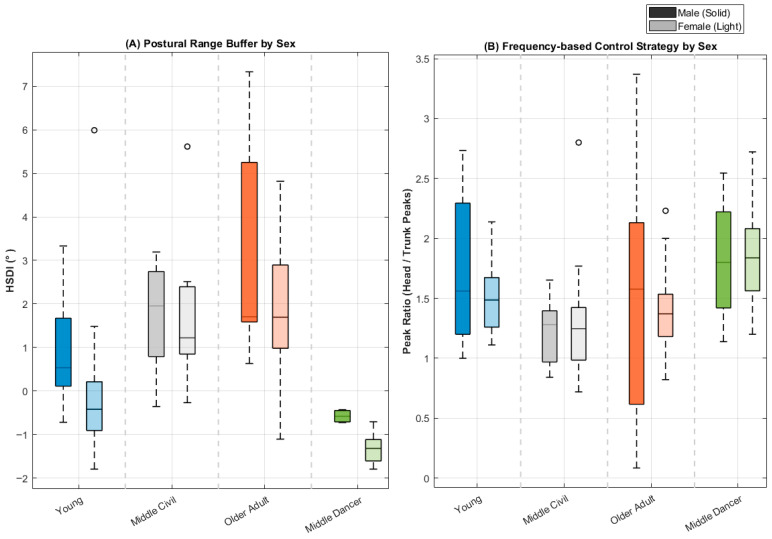
Sex-specific postural degradation profiles and the impact of rhythmic training across the lifespan. Boxplots illustrating the distribution of kinematic biomarkers among the four study cohorts, stratified by biological sex (Solid boxes: Males; Light/Transparent boxes: Females). (**A**) Postural Range Buffer (HSDI). Illustrates the spatial divergence in stabilization strategies. Note the narrower physiological limits in aging males (solid), which drive an early rigidification compared to the broader, more permissive spatial buffer observed in females (light). (**B**) Frequency-based Control Strategy (PR). Depicts the relative frequency of corrective micro-movements. The progressive decline in the male PR (particularly in the Middle-Aged Civil cohort) signifies a transition towards a rigid “Stiffening” strategy. In contrast, Middle-Aged Dancers successfully maintain a highly active, youth-like correction frequency regardless of sex, highlighting the neuroprotective effect of rhythmic training.

The comparative analysis revealed a distinct advantage for those with rhythmic expertise. As shown in Figure 3 and Table 1, the Middle-Aged Dancer group did not merely “maintain” stability but outperformed the Young Adult group, achieving a mean HSDI of −2.05° (vs. 0.30° in Young). This negative value reflects an active “Isolation Strategy” (similar to a gimbal mechanism), where the head’s ROM is dampened relative to trunk perturbations. In contrast, the Middle-Aged Civil group exhibited significantly higher positive values (3.01°), indicating early signs of head stability deterioration. To rigorously quantify this protective effect despite the smaller sample size of the dancer cohort (*n* = 10), effect size was calculated against the age-matched civil group (*n* = 20). The analysis yielded an exceptionally large effect size for HSDI (Cohen’s d = 2.20, *p* < 0.0001) and PR (Cohen’s d = 1.15, *p* = 0.005), statistically confirming that rhythmic training provides a powerful, highly reliable defense against age-related kinematic decay.

### 3.3. Validation and Multi-Axial Reliability

To ensure the clinical utility of the IMU-derived metrics, we performed a comprehensive validation against the Zebris force platform.

- Primary Validation: The lateral HSDI demonstrated a strong positive correlation with the total COP excursion area (r = 0.62, *p* < 0.001), confirming that higher segmental dissociation/stiffness directly reflects overall postural instability.
- Directional Consistency: Significant correlations were observed between IMU angular displacements and COP excursions in both the ML and anteroposterior (AP) axes (r > 0.60 for all tasks).
- Supplementary Data: Detailed cross-correlation matrices and the relationship between Y-axis (ML) and X-axis (AP) kinematic fluctuations are provided in Supplementary Materials (Table S1, Figures S1 and S2). This highlights that while AP movements are dominant during forward reaching, lateral HSDI serves as a more sensitive marker for the early detection of neural control degradation.
- Notably, while the Mileseey M120-B laser measurements were used to calculate absolute physical displacements (e.g., avg_angle_x and y), the HSDI’s reliance on raw kinematic streams proved to be independent of anatomical variations, supporting the feasibility of a streamlined, height-independent assessment.

### 3.4. Identification of the 55-Year “Kinematic Breakpoint”

**Figure 4:**
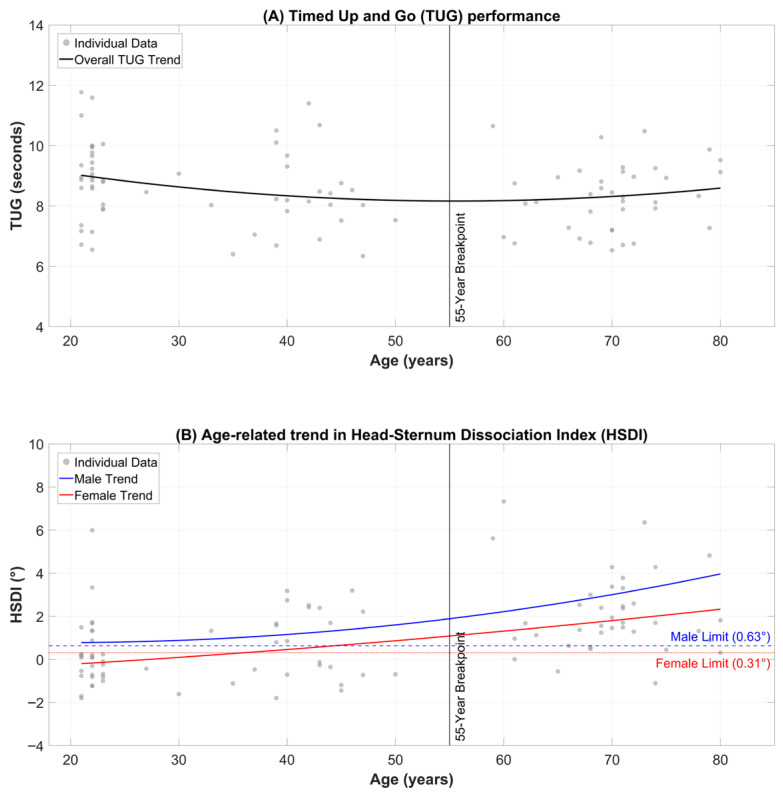
Age-Related Trends and Critical Breakpoint Progression. Comparison of functional mobility versus postural quality across the lifespan. (**A**) TUG performance: Functional mobility remains largely stable during the “Latent Phase” (green annotation), with a notable decline and increased variance observed only after the age of 55 (vertical line). (**B**) Age-related trend in HSDI: In contrast to TUG, kinematic stability shows early divergence. Note that the female trend (red solid line) indicates a progressive drift exceeding the specific instability threshold (0.31°, red dotted line) starting from age 45, revealing latent sensorimotor decline well before the 55-year functional breakpoint. The male trend (blue solid line) reflects a stiffer profile, remaining largely below the male limit (0.63°, blue dashed line) until the Older Adult cohort. Individual data points are shown in gray to highlight the population distribution.

The most striking finding of this study is the divergence between functional mobility (TUG time) and movement quality (HSDI) (Figure 4).

- Functional Mobility: No significant difference in TUG performance was found between the Young and Middle-Aged Civil groups (*p* > 0.05).
- The Breakpoint: Iterative ANOVA identified a significant breakpoint at age 55 (*p* < 0.01), where the transition from “Dissociation” to “Stiffness” strategies becomes generalized. Crucially, a sensitivity analysis confirmed that this age-related shift remained highly significant even when the dancer subgroup was completely excluded from the analysis.
- The Latent Deficit (Early Divergence): Crucially, the Middle-Aged Civil group (ages 40–54) showed HSDI values that were already significantly higher than the Young group (*p* < 0.05). This “early divergence” explicitly refers to the phenomenon where underlying kinematic stabilization strategies—particularly the sex-specific transition towards rigid or chaotic patterns—begin to collapse decades before macroscopic functional performance (TUG) shows any significant impairment.

### 3.5. Phenotyping Movement: The Kinematic Stability Matrix

**Figure 5:**
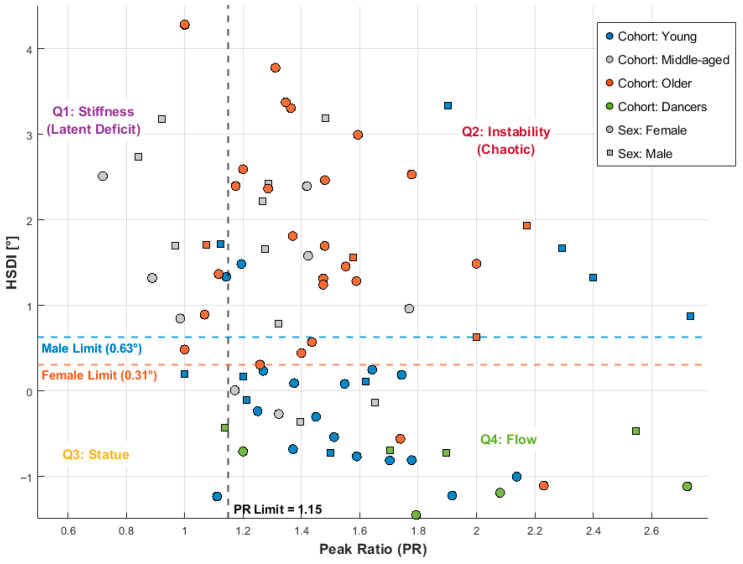
The Kinematic Stability Matrix (Sex-Specific Phenotypes). Scatter plot illustrating the relationship between Corrective Frequency (PR) and Instability Amplitude (Windowed HSDI) for the study cohort (N = 94). Individual data points are stratified by sex (Squares: Male; Circles: Female) and study group (Color coded). The quadrants categorize participants into four distinct stability phenotypes defined by a vertical functional coupling threshold (PR = 1.15) and horizontal sex-specific amplitude limits: Q1 (Stiffness): Low frequency (PR ≤ 1.15)/High amplitude (HSDI > Cut-off). Represents the “Latent Deficit” typical of middle-aged civilians, predominantly males. Q2 (Instability): High frequency (PR > 1.15)/High amplitude (HSDI > Cut-off). Represents the chaotic failure mode typical of older adults. Q3 (Statue) & Q4 (Flow): Efficient stabilization patterns populated by Dancers and Young Adults. Note on Sex-Specific Strategies: The optimal instability thresholds, established via ROC analysis, are distinct for males (Blue dashed line, 0.63°) and females (Red dashed line, 0.31°), confirming fundamentally different baseline control strategies between sexes.

The distribution of participants within the Kinematic Stability Matrix (Figure 5) provided a precise, quantitative mapping of postural control strategies. By analyzing the relationship between instability amplitude (HSDI) and corrective frequency (PR), we identified four distinct postural phenotypes.

Sex-Specific Baselines: Crucially, our ROC analysis (detailed in Supplementary Figure S3) revealed that optimal stability thresholds are sex-dependent. The physiological limit for instability was established at 0.63° for males and 0.31° for females, reflecting a stricter, “binary” control strategy in men (high baseline stiffness) versus a finer, more sensitive tuning in women. Based on these physiological baselines and the functional coupling threshold of PR = 1.15, participants fell into four zones. To address the uneven distribution across these quadrants, Table 2 details the demographic clustering, which directly reflects distinct biological aging phenotypes.

- Quadrant 1 (Stiffness—Latent Deficit): Characterized by low corrective frequency (PR ≤ 1.15) and elevated instability (HSDI > Cut-off). This zone represents a “freezing” strategy where individuals attempt to mask proprioceptive errors by rigidly locking the head–trunk segment. As seen in Table 2, this quadrant accumulates middle-aged and older individuals, confirming the presence of a “Latent Deficit”: their reduced micro-mobility reveals a brittle control strategy prone to sudden failure.
  Clinical Intervention: Due to the rigid co-contraction strategy, clinical management should prioritize a higher proportion of segmental mobilization, axial flexibility exercises, and sensory re-weighting therapies to break the maladaptive stiffening pattern.
- Quadrant 2 (Instability—Chaotic): Characterized by high instability amplitude (HSDI > Cut-off) and high corrective frequency (PR > 1.15). This phenotype captures the oldest demographic (mean age 65.5 years), representing the collapse of the compensatory stiffening mechanism. The sensorimotor system resorts to chaotic, high-frequency corrections that fail to stabilize the head.
  Clinical Intervention: As the primary deficit is uncompensated oscillation, interventions must focus on a higher proportion of segmental stabilization, core strengthening, and reactive balance training to restore controlled movement boundaries.
- Quadrants 3 & 4 (Statue & Flow—Protective Effect): These zones represent efficient stabilization. Q3 (“Statue”) represents minimal effort stability, populated almost exclusively by the youngest individuals (N = 3). Q4 (“Flow”) represents fluid, rhythmic adjustments (PR > 1.15, HSDI ≤ Cut-off). Notably, the vast majority of Young adults and 100% of the Middle-Aged Dancers clustered in Q4, maintaining youthful kinematic profiles.
  Clinical Intervention: For individuals in these highly efficient states, the primary recommendation is maintenance through rhythmic sensorimotor training (e.g., dance) and dynamic coordination exercises to preserve their optimal neuro-mechanical baseline.

### 3.6. Impact of Biological Sex on Kinematic Profiles

Our analysis of the complete study cohort (N = 94) revealed that biological sex fundamentally shapes the baseline for postural stability. While the mean instability amplitude (Windowed HSDI) appeared comparable between sexes in the Young cohort (*p* > 0.05), the physiological limits derived—rigorously derived from ROC analysis (Figure S3)—exposed distinct control strategies:

- Baseline Divergence: Young Males operated within a narrower physiological window, reflecting a high-stiffness “Binary” strategy, where even minor deviations trigger rigid correction. In contrast, Young Females exhibited a broader tolerance, confirming a more permissive “Continuous” control strategy due to naturally higher baseline flexibility.
- Adaptation and Failure (Aging Trajectories): This baseline difference dictated the trajectory of decline. As males age, they predominantly shift toward a “Rigid Stiffness” strategy (Q1), effectively “freezing” their movement to stay within their strict limits. Conversely, aging females, utilizing their wider physiological buffer, eventually progress directly into high-amplitude “Uncompensated Dissociation” (Q2), characterizing a distinct, chaotic failure mode.

### 3.7. Dynamic Adaptation in the Pilot Study

Straight walking and turning as a supplementary analysis within the nested pilot study, we investigated the system’s sensitivity to dynamic spatial load shifts, separating the straight walking and turning phases of the 10-participant healthy female cohort. During straight walking segments, the HSDI remained stable and reliable (ICC = 0.774, mean HSDI = 3.33°), reflecting robust mediolateral control during the rhythmic step cycle. In turning phases, where centrifugal forces introduced asymmetrical loading, the mean HSDI predictably increased (4.26°). Concurrently, the variance of the PR compensatory strategy immediately rose (ICC = 0.450), proving that the device can successfully detect individual-specific neuromotor adaptations forced by sudden directional changes during dynamic locomotion.

## 4. Discussion

### 4.1. The “Stiffening” Strategy: A Biomarker for Latent Deficit

The primary objective of this study was to validate the HSDI as a sensitive digital biomarker. Our findings confirm our initial hypotheses: (1) IMU-derived kinematic metrics demonstrate strong concurrent validity with gold-standard force platforms; (2) a statistical breakpoint in postural control emerges at age 55; and (3) rhythmic training acts as a powerful protective factor.

Our findings demonstrate that the HSDI, when combined with the PR of corrective micro-movements, identifies postural decay significantly earlier than traditional clinical assessments [1,2]. While the Middle-Aged Civil group maintained functional performance within normal limits on the TUG test, a significant portion of this cohort was already clustered in the “Stiffness” quadrant (Q1) of the Kinematic Stability Matrix. This discrepancy confirms previous observations that time-based metrics alone often suffer from a ‘ceiling effect’ and are often insufficient to capture the qualitative erosion of motor control [3,4]. This “stiffening” or “trunk-locking” strategy—characterized by a pathological reduction in relative corrective frequency (PR ≤ 1.15) and rigid head–trunk coupling—is a compensatory response to perceived instability [6]. According to Bernstein’s “Degrees of Freedom” theory, when the central nervous system faces a decline in sensory reliability, it freezes distal segments to simplify the control problem [20]. Our data reveals that this is predominantly a male strategy. By rigidly locking the head to the sternum, middle-aged males successfully “mask” their proprioceptive deficits during predictable tasks [7,8]. However, this rigidity is maladaptive: it reduces the axial flexibility required for rapid, reactive adjustments, turning the “Stiffness” quadrant (Q1) into a waiting room for future falls.

Crucially, a horizontal comparison with existing IMU-related indicators clarifies the unique advantages of our approach. While traditional metrics (such as isolated trunk tilt angle or head sway velocity) capture absolute dynamic sway, the HSDI specifically quantifies the relative neuro-mechanical uncoupling (rigidification vs. chaotic dissociation) between segments, allowing for the identification of latent deficits much earlier than absolute amplitude metrics alone.

### 4.2. Sex-Specific Trajectories: Binary vs. Continuous Control

A pivotal finding of this study is that postural aging is not a uniform process but follows distinct, sex-specific trajectories defined by different physiological baselines [11].

The Male “Binary” Strategy: Males exhibited a higher optimal threshold for overt instability (0.63°). This suggests a “Binary” control model dependent on high muscle tone and rigid coupling. The shift of Middle-Aged males into Quadrant 1 reflects an effort to maintain this binary state via “freezing.” However, in Older Adults, this rigid strategy often collapses, leading to sporadic but significant breaches of the 0.63° threshold.

It is important to note the statistical differences in diagnostic predictive power between sexes. The lower predictive accuracy observed in males (AUC = 0.70, Youden’s J = 0.30) compared to females (AUC = 0.83, Youden’s J = 0.65) is not a methodological flaw, but rather a mathematical reflection of the brittle nature of the ‘Binary’ strategy itself. Rigid co-contraction can mask underlying sensorimotor deficits highly effectively until a sudden, unpredictable point of failure occurs. Consequently, this sudden kinematic collapse is inherently more challenging to capture with a single static diagnostic threshold than the progressive, ‘Continuous’ deterioration observed in the female cohort.

The Female “Continuous” Strategy: Females operated with a lower, more sensitive diagnostic threshold (0.31°), reflecting a “Continuous” strategy that relies on finer, fluid micro-adjustments rather than rigid locking. While hormonal factors may be one of the potential regulatory mechanisms affecting this sensorimotor sensitivity [21], this baseline allows females to maintain stability without resorting to pathological stiffness. However, once this compensatory buffer is exhausted in advanced age, females tend to drift into the “Instability” quadrant (Q2), characterized by high-frequency, chaotic oscillations rather than rigidity.

These findings challenge the “one-size-fits-all” approach to geriatric screening, necessitating sex-normative diagnostic criteria.

### 4.3. The Neuroprotective Role of Rhythmic Expertise (Dance)

The most striking finding is the 100% success rate of Middle-Aged Dancers in maintaining kinematic profiles indistinguishable from the Young Adult cohort. While their age-matched civil peers (particularly males) gravitated towards the rigid “Stiffness” strategy, dancers consistently occupied the high-efficiency “Flow” (Q4) and “Statue” (Q3) quadrants. This suggests that long-term rhythmic training acts as a powerful neuroprotective factor. We propose that dance preserves the “Continuous” control strategy even in males, effectively overriding the biological tendency towards stiffness. By constantly integrating complex auditory, vestibular, and proprioceptive inputs, dancers maintain a high “motor reserve” [22]. As Teixeira-Machado et al. noted, dance functions as a multisystemic intervention that preserves white matter integrity and enhances neuroplasticity in areas dedicated to balance and coordination [23]. Unlike the “trunk-locking” mechanism seen in civilians, dancers utilize fluid, active stabilization of the head–sternum unit. This flexibility indicates a highly functioning Vestibulo-Collic Reflex (VCR) that remains responsive and adaptable, effectively pinning the dancer’s biological age decades below their chronological age [22].

### 4.4. Clinical Implications: Democratizing Biomechanical Screening

The integration of our sex-specific thresholds (0.63° for males, 0.31° for females) and the PR = 1.15 into the Kinematic Stability Matrix offers a robust framework for a new generation of screening tools. Current fall-prevention strategies typically focus on the “frail” population (aged 65+), by which point many sensorimotor deficits are irreversible. Our data, however, reveals that the “Latent Deficit”—the pathological migration to the Stiffness quadrant—is detectable in middle-aged civilians well before functional failure occurs.

These findings advocate for a paradigm shift in primary care: moving high-precision biomechanics out of the laboratory and into general practice. By utilizing a minimalist setup of low-cost, wearable IMU sensors [9,13,24], clinicians can now identify the neural effort required to maintain function, which standard tests like the TUG miss [10,12]. A key advantage for clinical operability is that the HSDI utilizes angular displacement data processed internally by the IMU’s built-in Kalman filters. This provides robust, ready-to-use spatial values directly from the hardware, eliminating the need for complex, error-prone software post-processing (such as the integration of raw acceleration or velocity data). Combined with our standardized dual-sensor placement (forehead and manubrium sterni), this hardware-level fusion allows clinicians to perform a rapid, highly operable 30-s clinical assessment. Detecting these deficits early offers a proactive solution to the significant healthcare burden associated with trauma rehabilitation. Notably, epidemiological data confirms that the incidence of traumatic injuries significantly increases around the age of 55—mirroring the kinematic breakpoint identified in our study—further underscoring the urgency of mid-life screening [25]. Ultimately, this distinction allows clinicians to move beyond generic fall-prevention advice, prescribing targeted mobilization for the “stiff” male phenotype versus core stabilization for the “unstable” female phenotype.

### 4.5. Limitations and Future Directions

While this study provides compelling evidence for the use of HSDI in early postural screening, several limitations must be acknowledged.

First, the sample size of the Middle-Aged Dancer group (N = 10) is relatively small, though the effect size is mathematically substantial. Future studies should expand this to include other disciplines (e.g., martial arts) to assess if the “anti-stiffness” effect is unique to dance.

Second, the current assessment utilized instrumented functional reach tasks (iFRT, iLRT) to evaluate the limits of stability. While these quasi-dynamic tasks are foundational for assessing anticipatory postural adjustments and maximal excursion, they do not fully capture the continuous dynamic complexities of daily living. Future research should focus on validating the HSDI during continuous dynamic tasks (such as walking and turning), as well as seated postural control assessments, to further expand its clinical applicability and determine if the “Stiffening” strategy persists during locomotion [13,26,27].

Third, while we incorporated hormonal shifts as a theoretical factor explaining the divergence in aging trajectories [21], we did not perform direct biochemical measurements. Future multidisciplinary studies combining kinematic tracking with comprehensive endocrine profiling could provide direct evidence of how hormonal attrition triggers the collapse of compensatory motor strategies.

Furthermore, while the data-driven identification of the 55-year kinematic breakpoint is statistically highly significant within our recruited sample (N = 94) and survived rigorous sensitivity analyses, we acknowledge the inherent risk of sample-specific overfitting. This 55-year threshold should be viewed as a strong, preliminary clinical indicator of latent decline. To fully validate this specific chronological milestone for global clinical screening, external validation in larger, multi-centric, and demographically diverse cohorts is required.

Although our analysis reveals distinct age-related kinematic profiles, the cross-sectional design inherently limits our ability to definitively establish individual causal trajectories. Terms used throughout this study—such as ‘drift,’ ‘shift,’ or ‘transition’—are intended to describe generalized, population-level differences across age cohorts rather than individually tracked longitudinal changes. While the cross-sectional evidence for these distinct neuro-mechanical pathways is robust, future prospective cohort studies are necessary to confirm the exact temporal progression from ‘Binary Stiffness’ to overt clinical collapse.

## 5. Conclusions

This study establishes the HSDI as a sensitive, kinematic biomarker capable of identifying postural decline decades before it becomes apparent through standard clinical assessments. By utilizing a scalable configuration of wearable IMU sensors, we successfully mapped distinct postural profiles, ranging from youthful, dynamic stability to the compensatory strategies that precede overt functional failure.

Our results establish three critical findings:

- The Latent Deficit: Sensorimotor erosion begins as early as the fourth decade of life. Crucially, this manifests differently between sexes: males predominantly adopt a “Stiffness” strategy (Quadrant 1) to mask instability, while females rely on a wider physiological buffer before exhibiting chaotic oscillation. This confirms that traditional time-based tests (like TUG) are “blind” to the qualitative cost of maintaining stability.
- Sex-Specific Physiological Baselines: We identified distinct control strategies defined by quantitative limits. Males operate under a higher threshold (0.63°) reflecting a rigid “Binary” control model, whereas females utilize a “Continuous” strategy marked by a lower, more sensitive threshold (0.31°) that relies on fluid micro-adjustments rather than pathological stiffness. Recognizing these distinct baselines is essential for accurate geriatric screening, necessitating sex-normative diagnostic criteria.
- Neuroprotection through Rhythm: The consistent ability of Middle-Aged Dancers in our sample to maintain youthful “Flow” kinematics demonstrates that postural decline is not inevitable. Recreational rhythmic training acts as a powerful neuroprotective intervention, effectively decoupling biological age from chronological age by preserving reflex efficiency and axial flexibility.

In conclusion, the HSDI offers a cost-effective and objective screening tool for primary care. By identifying individuals in the “Stiffening” phase (males) or the “Drifting” phase (females), clinicians can implement targeted, sex-specific interventions—such as rhythmic movement therapies—to preserve functional independence and rewrite the trajectory of active aging.

## Figures and Tables

**Table 1:** Participant demographics and primary outcome measures.

Group	N	Age (Years)	TUG (s)	HSDI (Deg)	PR
Young	30	23.1 ± 0.8	8.96 ± 1.3	0.30 ± 3.0	1.56 ± 0.42
Middle-Dancer	10	42.9 ± 10.2	7.80 ± 1.3 *	−2.05 ± 1.8 *	1.85 ± 0.51 *
Middle-Civil	20	45.4 ± 7.8	8.73 ± 1.3	3.01 ± 2.9 *	1.30 ± 0.45
Older Adult	34	72.1 ± 4.7	8.28 ± 1.1 *	4.07 ± 3.6 *	1.42 ± 0.55

Participant demographics and primary outcome measures. Data are presented as Mean ± Standard Deviation (SD). TUG: Timed Up and Go test; HSDI: Head–Sternum Dissociation Index; PR: Peak Ratio (ratio of head to sternum corrective micro-movements identified by the ZigZag algorithm). Note that the Middle-Aged Civil group exhibits the lowest PR (1.30), indicating a shift towards a rigid “Stiffening” strategy (reduced head-independence), whereas Middle-Aged Dancers maintain a highly uncoupled, active stabilization pattern (PR = 1.85). (*) denotes significant difference (*p* < 0.05) compared to the Young group, except for PR where it indicates significant difference compared to the Middle-Aged Civil cohort.

**Table 2:** Distribution of Participants Across Kinematic Phenotypes (N = 94).

Quadrant (Phenotype)	Kinematic Profile	Total (N)	Female (*n*)	Male (*n*)	Mean Age (Female/Male)
Q1 (Stiffness/Latent Deficit)	PR ≤ 1.15, HSDI > Cut-off *	18	11	7	54.2/48.4 yrs
Q2 (Instability/Chaotic)	PR > 1.15, HSDI > Cut-off *	37	24	13	65.5/44.5 yrs
Q3 (Statue)	PR ≤ 1.15, HSDI ≤ Cut-off *	3	1	2	22.0/24.5 yrs
Q4 (Flow)	PR > 1.15, HSDI ≤ Cut-off *	36	27	9	33.6/34.3 yrs

PR, Peak Ratio; HSDI, Head–Sternum Dissociation Index. The classification thresholds were established at PR = 1.15 (15% physiological margin of error). * The HSDI Cut-off was applied strictly based on sex-specific ROC analyses: >0.63° for males and >0.31° for females.

## Data Availability

The data presented in this study are available on request from the corresponding author.

## Supplementary Materials

The following supporting information can be downloaded at: https://www.mdpi.com/article/10.3390/s26072125/s1, Figure S1: Anterior–Posterior (Sagittal) synchronization between the height-weighted IMU average and the Zebris COP displacement; Figure S2: Medio-Lateral (Coronal) synchronization between the height-weighted IMU average and the Zebris COP displacement; Table S1: Correlation coefficients (Pearson’s r) between individual IMU sensors; Figure S3: Sex-Specific ROC Analysis for Latent Deficit Detection.

## Abbreviations

The following abbreviations are used in this manuscript:

ANOVA	Analysis of Variance
AP	Anteroposterior
AUC	Area Under the Curve
COP	Center of Pressure
FRT	Functional Reach Test
HSDI	Head–Sternum Dissociation Index
iFRT	Instrumented Functional Reach Test
iLRT	Instrumented Lateral Reach Test
IMU	Inertial Measurement Unit
iTUG	Instrumented Timed Up and Go
LRT	Lateral Reach Test
ML	Medio-Lateral
PR	Peak Ratio
ROC	Receiver Operating Characteristic
ROM	Range of Motion
SD	Standard Deviation
TUG	Timed Up and Go
VAS	Visual Analogue Scale
VCR	Vestibulo-Collic Reflex
