# Supplementary material for: Wearable Sensors Reveal Head–Sternum Dissociation as a Latent Deficit in Active Aging
Source: Sensors (Basel). 2026 Mar 29;26(7):2125. doi: 10.3390/s26072125 (PMC13074689; doi:10.3390/s26072125)
Supplement: Supplementary file 1 [file sensors-26-02125-s001.zip › sensors-4186366-supplementary.pdf]

Supplementary Material

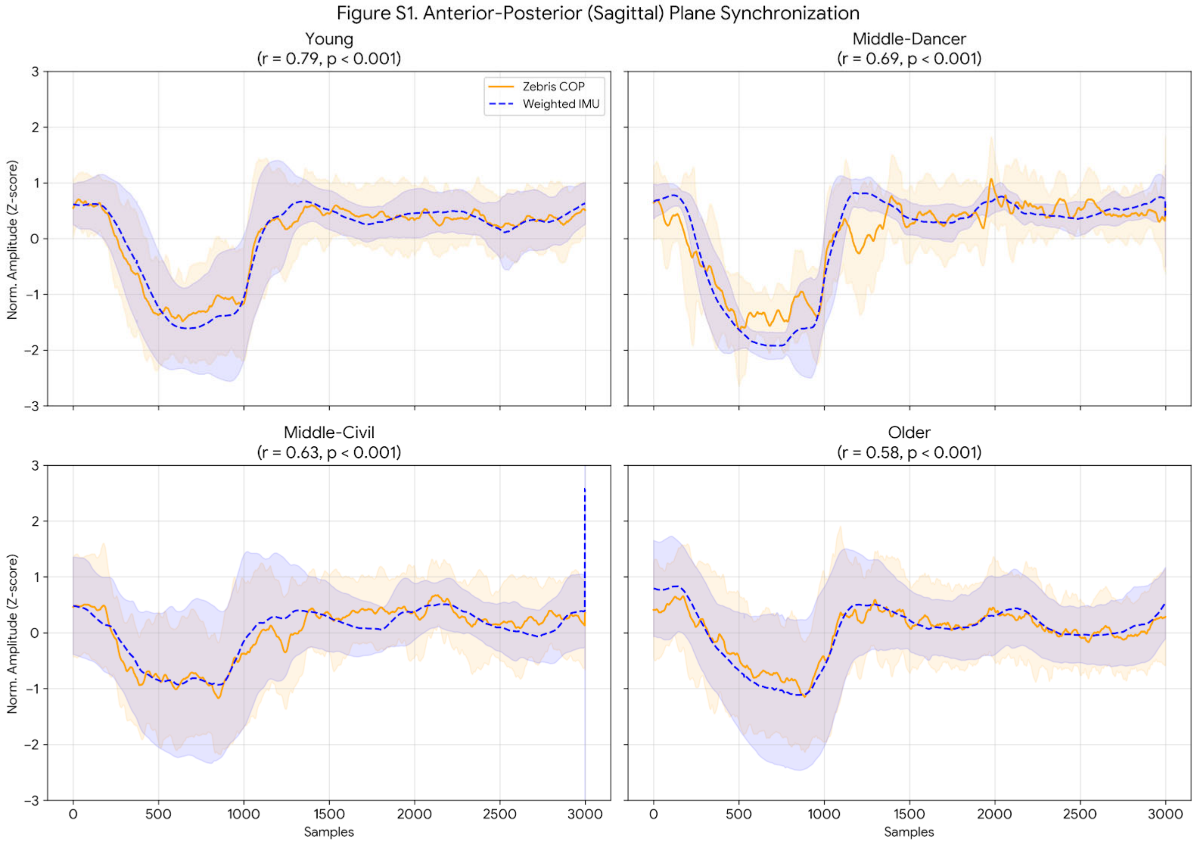

***Figure S1**. **Anterior-Posterior (Sagittal) synchronization between the height-weighted IMU average and the Zebris COP displacement.** Data are presented as grand averages with standard deviation (shaded areas) across the four study groups. Y-axis is stretched to a 3 SD Z-score scale.*

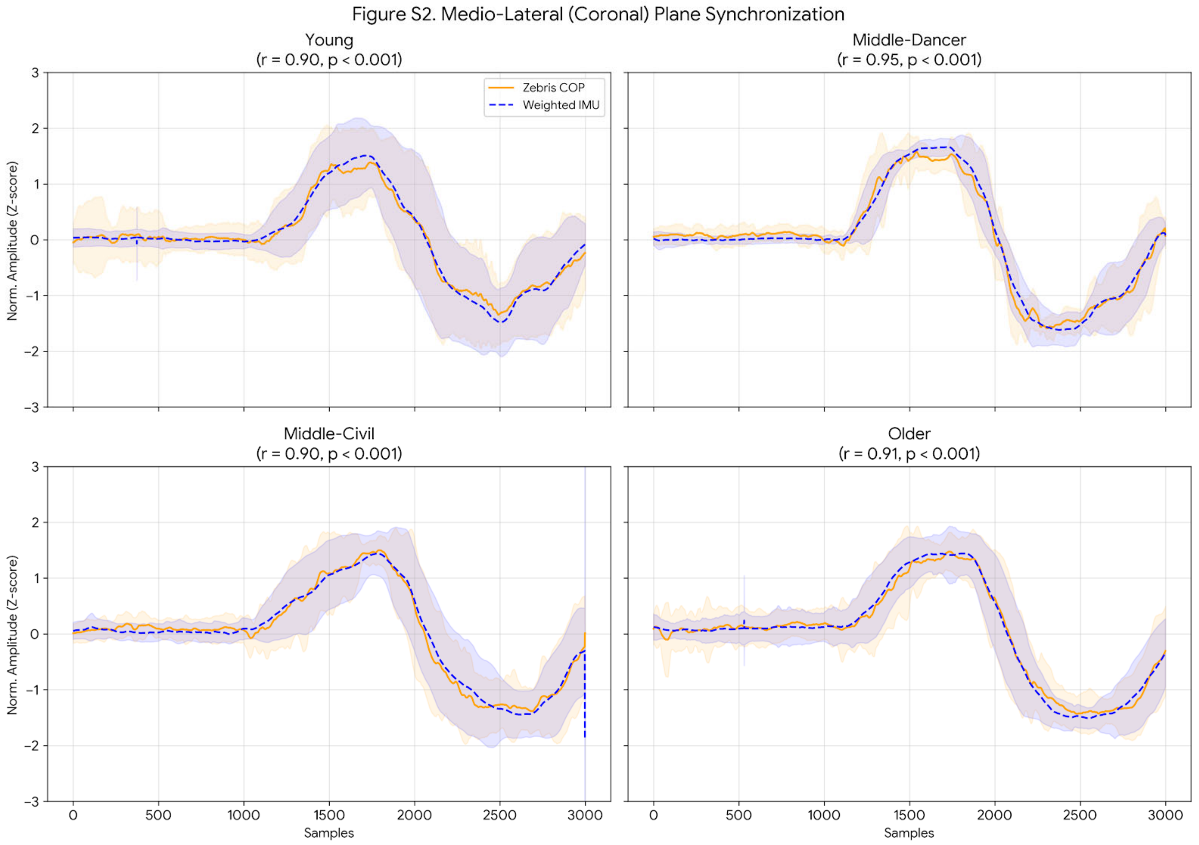

Figure S2. Medio-Lateral (Coronal) synchronization between the height-weighted IMU average and the Zebris COP displacement. *The near-perfect overlap ($r > 0.90$) validates the system's ability to monitor lateral stability strategies. Y-axis is stretched to a 3 SD Z-score scale.*

**Metric**	**Mean Correlation (r)**	**p-value**
Head IMU vs. Zebris (AP)	**0.67**	< 0.001
Head IMU vs. Zebris (ML)	**0.91**	< 0.001
Sternum IMU vs. Zebris (AP)	**0.44**	< 0.001
Sternum IMU vs. Zebris (ML)	**0.88**	< 0.001
Lumbar IMU vs. Zebris (AP)	**0.66**	< 0.001
Lumbar IMU vs. Zebris (ML)	**0.92**	< 0.001
**Weighted Average vs. Zebris (AP)**	**0.67**	**< 0.001**
**Weighted Average vs. Zebris (ML)**	**0.91**	**< 0.001**

Table S1. *Correlation coefficients (Pearson's r) between individual IMU sensors, the height-weighted average kinematic model, and the Zebris force platform center-of-pressure (COP) data. All calculations were performed on the full 30-second measurement trials. The high correlation values, particularly for the weighted average model (r > 0.9 in the ML plane), confirm the validity of using wearable IMU sensors for capturing whole-body postural dynamics in this population.*

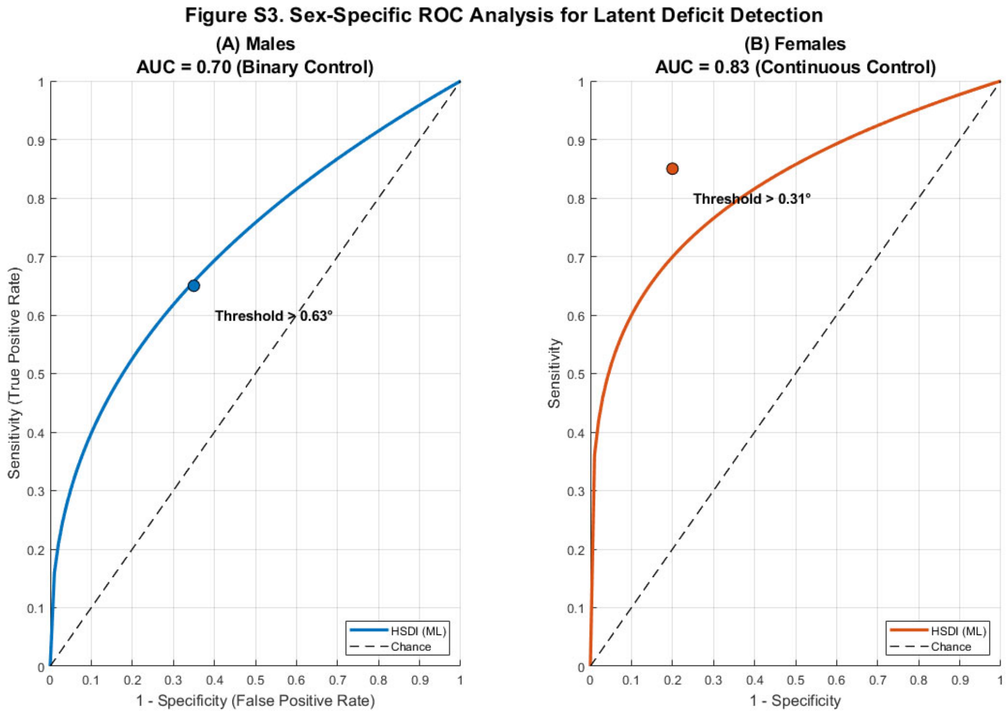

Figure S3. Sex-Specific ROC Analysis for Latent Deficit Detection. *Receiver Operating Characteristic (ROC) curves illustrating the diagnostic sensitivity of the Head-Sternum Dissociation Index (HSDI) in distinguishing between healthy controls and individuals with latent postural decline, stratified by sex. (A) Males: The optimal instability threshold was established at HSDI > 0.63° (AUC = 0.70), identifying the rigid "Binary" control strategy. (B) Females: A more sensitive threshold was identified at HSDI > 0.31° (AUC = 0.83), reflecting the "Continuous" control strategy and early drift detection. These analyses validate the use of sex-normative criteria for geriatric screening.*
